# How Retting Could Affect the Mechanical Behavior of Flax/Epoxy Biocomposite Materials?

**DOI:** 10.3390/ma16072929

**Published:** 2023-04-06

**Authors:** Mohamed Ragoubi, Morgan Lecoublet, Mehdi Khennache, Leonard Ionut Atanase, Christophe Poilane, Nathalie Leblanc

**Affiliations:** 1 UniLaSalle, Unité de Recherche Transformation et Agro-Ressources, VAM2IN (ULR 7519 UniLaSalle—Université d’Artois), 76130 Mont-Saint-Aignan, France; 2 Faculty of Medical Dentistry, Apollonia University of Iasi, 700511 Iasi, Romania; 3 Academy of Romanian Scientists, 050045 Bucharest, Romania; 4 Normandie Université, Université de Caen Normandie (UNICAEN), 14000 Caen, France

**Keywords:** flax morphology, flax fibers, retting effect, biobased composite, mechanical performances

## Abstract

This study focuses on the retting effect on the mechanical properties of flax biobased materials. For the technical fiber, a direct link was established between the biochemical alteration of technical flax and their mechanical properties. In function of the retting level, technical fibers appeared smoother and more individualized; nevertheless, a decrease in the ultimate modulus and maximum stress was recorded. A biochemical alteration was observed as the retting increased (a decrease in the soluble fraction from 10.4 ± 0.2 to 4.5 ± 1.2% and an increase in the holocellulose fractions). Regarding the mechanical behavior of biocomposites manufactured by thermocompression, a non-elastic behavior was observed for the tested samples. Young moduli (E1 and E2) gradually increased with retting. The retting effect was more pronounced when a normalization was performed (according to the fiber volume and porosity). A 40% increase in elastic modulus could be observed between under-retting (−) and over-retting (+). Moreover, the porosity content (Vp) increased overall with fiber content. Setup 3, with optimized processing parameters, was the most desirable processing protocol because it allowed the highest fiber fraction (*Vf*) for the lowest Vp.

## 1. Introduction

For the development of ecofriendly and more efficient materials, research and industrial focus on the use of natural resources as an alternative to the petrol-based materials is highly favored. In this context, flax fiber-reinforced composites have attracted much attention from industrial and researchers. Compared to conventional fiber, flax fibers possess a lower price, relatively high specific strength and modulus, good sound absorption and heat insulation performance, recyclability and availability [1,2]. Despite these advantages, some drawbacks restrain the use of natural fibers as reinforcement in key industrial applications (limited stress, adhesion to certain matrix, as well as batch-to-batch variability) mostly due to the retting step [3,4]. The retting is a crucial step in the preparation of flax fibers. In France, flax crops are dew-retted to let the micro-organisms degrade the middle lamella in order to individualize the fibers [5]. The retting for textile applications is very well known and optimized [6]. Further efforts are needed to determine the optimal retting process and an optimal polymer/fiber interface without reaching the degradation of the lignocellulosic fiber itself. Moreover, researchers have studied this issue and no consensus has been reached yet. For example, Martin et al. studied Alizée flax variety with different retting time [7]. They highlighted that tensile properties of single flax fibers depend on retting time. The Young’s modulus and tensile strength of single flax fibers increased with the retting time, without notable phenomenon on the elongation at break. According to the authors, the reason for this behavior was complex but presumably related to the removal of low mechanical products with retting. Bourmaud et al. have also studied by nanoindentation as well by AFM the same retted flax batch [8]. The elastic modulus increases by 17 and 23%, respectively, between the shortest and the longest retting time. These statistically relevant results were associated with an alteration of the flax cell structure. However, Alix et al. tested the influence of retting time on the tensile properties of unit flax fiber with 10 mm gauges [9]. They noted that no significant difference is observable between the different retting times. Placet et al. even observed a degradation of the mechanical performances of industrial hemp fiber subjected to a longer retting time due to a more advanced degradation of the unit fibers by the microorganisms [10].

Considering their different botanical origins, varieties, and variability, many studies [11,12,13] have been carried out on elementary flax fiber, but the potential of technical flax fibers as reinforcement was not entirely investigated until now [14,15]. Under industrial conditions, the plant fibers constituting the composites are also arranged in technical fibers in addition to the unit fibers arrangement. Technical flax fibers consist of a combination of elementary fibers interconnected at their interface by pectin [6,14]. They are obtained by scutching flax stems and hackling flax bundles. When the stems are scutched, flax shives and flax tows whose fibers are weakly bound together are recovered. An additional separation step through these weak interfaces is performed, producing technical fibers with a higher fineness and fewer cells in the bundles than simply scutched fibers [16]. Given the multiwall complex structure of the technical flax fibers, their performances are driven by certain physical properties (density, section size, etc.) and particularly their retting quality. According to some studies [12,13], retting contributes significantly to enhance and improve the Young’s modulus composites based on these fibers, their ultimate stress and reduce their elongation at break. The cellulose increase is responsible for the observed improvement and is primarily responsible for the plant stiffness. This improvement has been also observed in polypropylene-short fibers biobased composites made by injection [7]. Numerous papers have investigated the mixture between long fibers and an epoxy matrix, which has a good balance of mechanical properties. Fibers are either woven (twill, satin, plain, etc.) or unidirectional. According to the law of mixtures, the unidirectional fibers provide an elastic modulus between the matrix and the fiber one. For a fiber fraction (*V_f_*) of 50%, the authors of [17,18,19] reported Young’s moduli ranging from 27.2 to 39 GPa and ultimate stresses ranging from 296 to 408 MPa.

This highlights the importance of understanding the flax behavior during retting in order to promote the integration of flax in biobased composites. In this context, this study aims to focus on the way in which retting could affect the mechanical performance of flax-based biocomposites. An advanced analysis of technical flax fibers associated to three different retting levels: textile-optimized (0), under-retting (−) and over-retting (+) with the goal to analyze the impact of retting time on the mechanical properties of technical fibers and the derived technical flax biobased composites. The originality of this work lies in the idea of studying the effect of the technical flax fibers, not of the single fiber.

## 2. Materials and Methods

### 2.1. Raw Materials

**Figure 1:**
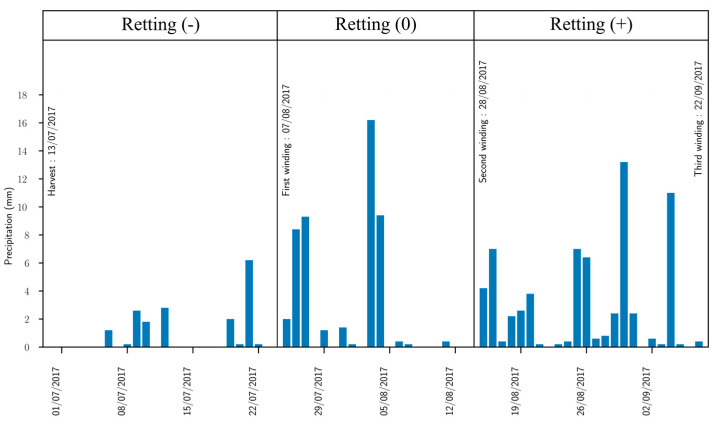
Flax cultivation conditions.

Epoxy XB3513 resin and Aradur 5021 polyimide crosslinker were used as the matrix polymer and provided by Vitech Composites (Sainte-Maure-de-Touraine, France). For the plant fiber, flax of the Bolchoi variety was used. It was grown in Romilly La Puthenay, Normandy, France. The seeding, harvesting and retting conditions are summarized in Figure 1. The retting dates were set at 7 August 2017 (W1), 28 August 2017 (W2) and 22 September 2017 (W3). The flax was scutched, but no hackling step, nor thermal, chemical, or physical preparation was carried out. Further cultivation conditions were described in a previously published paper [5]. Once the flax was scutched, it received a pre-impregnation step by the Vitech Composite society. The average fiber mass fraction was given as 50% ± 3. The matter was then kept in the laboratory in a −18 °C room.

### 2.2. Flax Composite Preparation

For the composite preparation, flax veils were impregnated with the epoxy resin to form prepregs sheets. The impregnation step was conducted on non-woven UD flax bands with a 110 g·m^−2^. The water-diluted epoxy was then sprayed on the flax bands and finally pre-cured at 120 °C. The resulting flax–epoxy prepregs presented a fiber content of Wf = 50% ± 3. After that, flax–epoxy prepregs were cut into 30 cm squares. Then, they were stacked in a UD pattern and placed between two metal plates. Two sheets of PTFE were added to facilitate the demolding step. At the end, we obtained biobased composite plates with an average thickness of 493 ± 31 µm and average density of 1.262 ± 0.024 g·cm^−3^. The thermocompression molding process was tested according to parameters summarized in Table 1. Setup 1 was the standard protocol used by the industrial partner, and Setup 3 was the optimized protocol proposed during this study. At the end of the demolding step, the composites obtained had an average thickness of 479 (±64) µm and a weight of 655 (± 42) g·m**^−^**^2^.

The determination of the fiber content (*V_f_*) and the porosity content (V_p_) of our biobased composites was achieved by the combined use of TGA and pycnometer.

### 2.3. Tensile Test for Flax Fibers

Mechanical tests were carried out by using an MTS Criterion 43 tensile machine. Tested lengths ranged from 14 to 100 mm, with 2 mm increments (thirty-three samples by retting mode). The displacement rate was set at 1 mm·min^−1^. It is important to note that we used a new method [5] to determine the average cross-section of each technical fiber by weighing and obtaining accurate knowledge of sample density.

### 2.4. Tensile Test for Biobased Composite Materials

Tensile tests were carried out on a Shimadzu traction machine with a 50 kN capacity load and self-tightening jaws. The displacement rate was fixed at 2 mm·min^−1^ and carried out at 23 °C and 65% HR. Specimens prepared according to ISO 527 were cut by a laser beam with a cutting speed of 35 mm·s**^−^**^1^. Flax–epoxy plates were glued at the area pinched by the jaws to avoid early breaks. For each material, five samples were tested.

### 2.5. Dynamic Mechanical Analysis

Dynamic mechanical analysis (DMA) was carried out in 3-point bending in a Netzsch DMA 242 system. Sample dimensions were 40 × 10 × 0.5 mm. The heating setup consists of a temperature slope from 30 to 130 °C at 3 °C·min**^−^**^1^. During this analysis, the oscillation was strain-controlled, with a dynamic amplitude up to 30 µm and a force range of 4 ± 4 N. Measurements were conducted from 0.1 to 1 Hz frequencies.

### 2.6. Biochemical Analysis

The chemical composition (cellulose, hemicelluloses and lignin) on the flax samples was performed according to the AFNOR standard (XPU44-162), derived from the Van Soest method [20]. Tests were carried out on 1 g of flax sample using a FOSS fiber device. It allowed us to determine the biochemical fractions of flax sample using different solvents. The results for the different chemical parameters were expressed in relation to the dry matter. The analytical dry matter was measured in a ventilated oven at 105 °C for 48 h. Cellulose, hemicelluloses and lignin were determined from the neutral detergent insoluble residue (NDF), acid detergent insoluble residue (ADF) and acid detergent and H_2_SO_4_ (%) (*w*/*w*) insoluble residue.

## 3. Results

### 3.1. Mechanical Properties of Technical Flax Reinforcements

**Figure 2:**
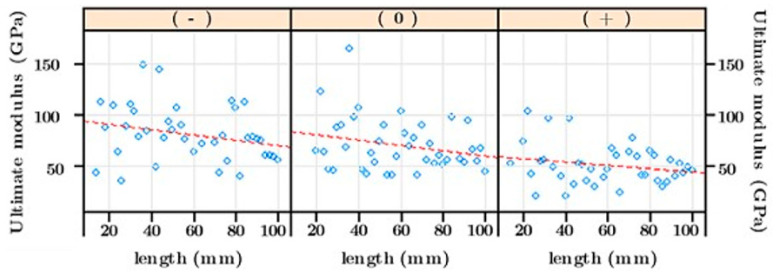
Ultimate modulus of flax fibers.

Figure 2 summarizes the mechanical properties of technical flax fibers for different gauge lengths (from 14 to 100 mm with a 2 mm step). The mean value of the results being not appropriate in our case study, it was extrapolated by linear regression to 0 mm and 100 mm in order to deduce the theoretical values for short (0 mm) and long technical flax fiber (100 mm), respectively. For the fiber length closer to 0 mm, mechanical performances were similar to those of single elementary fiber. In fact, mechanical performances of the 100 mm technical fiber were lower compared to that of theoretical elementary fiber of 0 mm. This may be due to the upper scale of the technical fiber containing more bundle/bundle interfaces (and fiber/fiber interfaces) linked by pectins, which are well known for their weak mechanical properties [21,22].

**Figure 3:**
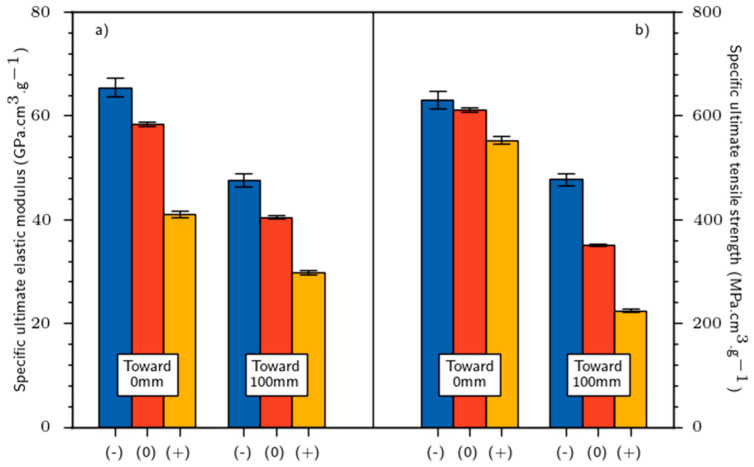
Tensile tests for flax fibers: (**a**) specific elastic modulus et (**b**) specific tensile strength.

Moreover, the elastic modulus and tensile strength decreased when the retting level was more pronounced (Figure 3). The ultimate elastic modulus decreased from 96.1 to 60.1 GPa for the theoretical elementary fiber and decreased from 69.9 to 43.6 GPa for the 100 mm technical fiber. The decrease in the tensile strength and elastic modulus with the increase in the retting level has been observed by [23,24] for technical fibers, but there is no consensus yet. Requile et al. [25] showed an improvement in the elementary fiber modulus with retting. However, other studies [9,26] reported no significant impact of retting on mechanical answer of technical and single fiber, respectively. The same negative impact of retting on the specific mechanical properties of flax fiber were noted. The specific elastic modulus decreased from 65.4 to 41.1 GPa/g·cm**^−^**^3^ for the theoretical elementary fiber and decreased from 47.6 to 29.8 GPa/g·cm**^−^**^3^ for the 100 mm technical fiber. The specific tensile strength varied from 631 to 553 MPa/g·cm**^−^**^3^ and from 477 to 224 MPa/g·cm**^−^**^3^ for the theoretical elementary fiber and the 100 mm technical fiber, respectively. Similar results have been reported by Zhu et al. [27]. Whatever the retting level, we should mention that the flax density remained stable (average density of 1.46 ± 0.04).

### 3.2. Mechanical Properties of Flax Biobased Materials

**Figure 4:**
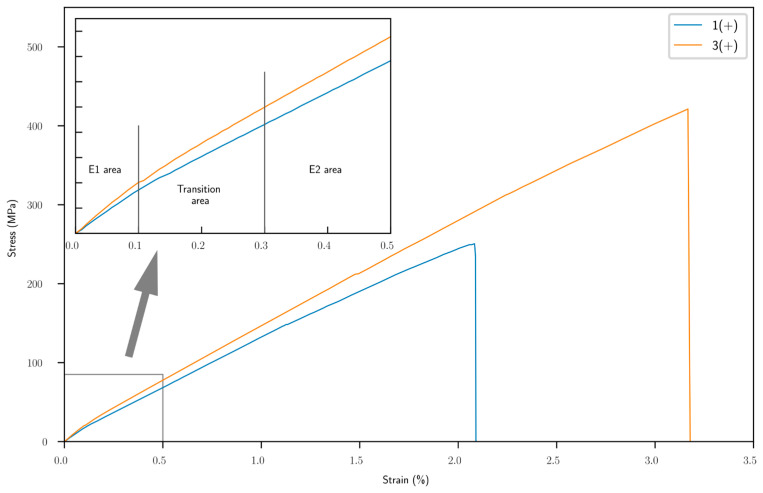
Tensile stress–strain curves of biobased composites; zoom on the small deformation area (until 0.5%). Blue line is 1 (+), orange line 3 (+).

Figure 4 shows typical tensile stress vs. strain curves for our biobased composite materials. We observed a non-linear stress–strain curve based on the raw values, unlike glass or carbon composites. These observations are also confirmed in the literature [28,29]. We noticed mainly three areas with different behavior:

(i) Area 1 (0–0.1% deformation): an elastic transition. This area is used to calculate the E1 modulus (modulus of small deformations).
(ii) Area 2 (0.1–0.3% deformation): a plastic transition and attributed to quick rearrangement of crystalline cellulose microfibrils between them and leading to a reduction in the stiffness between area 1 and area 3.
(iii) Area 3 (over 0.3%): the second elastic transition. The E2 modulus (for large deformations) was calculated between 0.3 and 0.5% of deformation.

**Figure 5:**
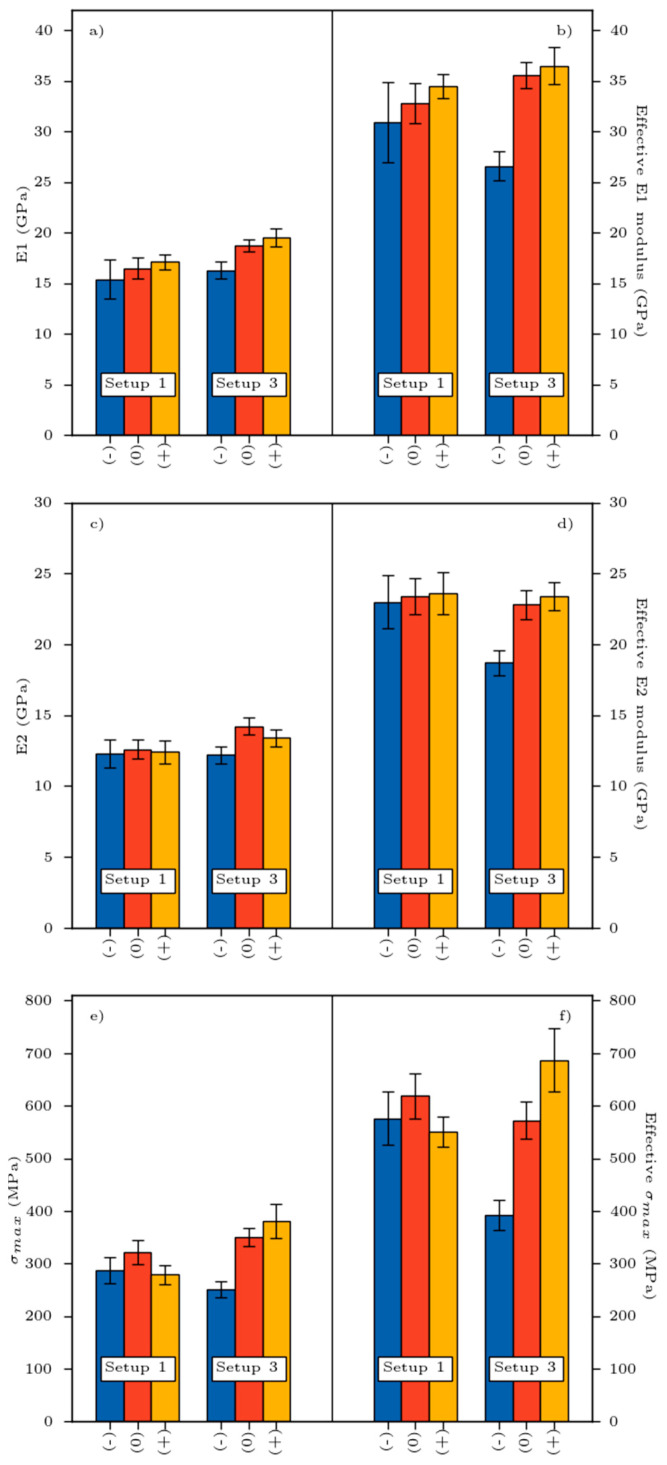
Variation of mechanical properties: (**a**,**b**)—E1 modulus, (**c**,**d**)—E2 modulus, (**e**,**f**)—maximal stress, in function of retting and process.

The variation in Young moduli (E1, E2) and tensile strength according to the influence of retting and setup is illustrated in Figure 5a–d. On the one hand, the elasticity performances are better as the retting increases. The higher the retting level, the higher the modulus E1. Regarding E2 modulus, it varied between 11.1 (±0.4) and 14.2 (±0.6) GPa, 25% lower than Young’s modulus (E1). On the other hand, setup 3 seems helpful to provide the best elasticity properties (around 19.5 GPa) compared to the setup 1.

For better understanding, we present the standardized mechanical properties of the tested materials (mechanical properties related to *V_f_*) in Figure 5. We considered that the mechanical properties of our biobased materials comply with the rule of mixture obtained by Equation (1).

(1) $$E_{C}=\eta_{0}\times \eta_{1}\times V_{f}E_{f}+V_{m}E_{m},$$

where *E_c_* is composite Young’s modulus; *E_f_* is longitudinal Young’s modulus of the fiber; *E_m_* is matrix Young’s modulus; *V_m_* is volumic matrix content; *V_f_* is volumic fiber content; *η*_0_ is fiber orientation efficiency (=1); *η*_1_ is fiber length efficiency (=1).

From Figure 5, it is more evident that retting positively impacted the mechanical performance of biobased composite materials. Regardless of the setup used, a 40% increase in modulus can be observed between retting (−) and (+). The higher the retting, the higher the fiber individualization process which is responsible for this improvement. E1 modulus displays no difference from a statistical point of view, although the best result seems to have been achieved for biobased composite 3 (+).

### 3.3. Dynamic Mechanical Properties

**Figure 6:**
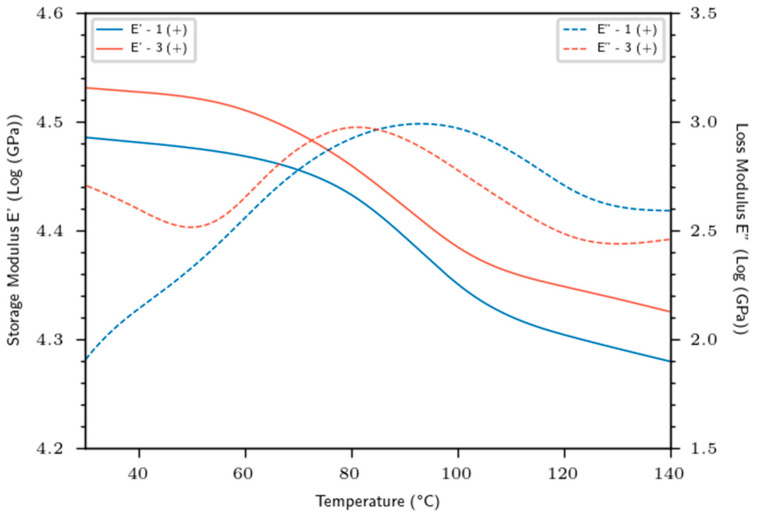
Dynamic mechanical properties of flax biobased materials.

Figure 6 shows typical viscoelastic properties (E′, E″) versus temperature of different biobased composites manufactured with the (+) retting. The frequency was fixed at 1 Hz. We distinguished an elastic behavior of flax biobased materials until 60 °C, followed by a rubbery behavior for T > 60 °C. The storage modulus (E′) is more important for flax composites made with setup 3 than that made fir for setup 1 and is correlated with the higher fiber content and a lower porosity rate observed when setup 3 is used. Whatever the setup and the composites materials used, a softening behavior with temperature was noticed due to a general softening of the epoxy matrix, allowing a greater molecular mobility of the macromolecular chains [30]. The sharp drop between 60 and 110 °C could be associated with the α relaxation caused by the glass transition of the epoxy matrix. Qi et al. [31] discovered a modulus of 2.5 GPa for the neat epoxy at room temperature and at 1Hz, demonstrating the key role of flax reinforcements in terms of stress transfer and elastic behavior. As the temperature increases, the epoxy softening becomes more pronounced and the E’ modulus continues to decrease up to 140 °C.

Regardless of temperature and frequency, a double positive effect of both retting and processing parameters on the storage modulus was determined. At mild conditions (40 °C, 1 Hz), E′ increased by 13% (*p*-value = 2.70 × 10^−2^) from setup 1 to setup 3. Furthermore, E’ increased by 15% (*p*-value = 1.60 × 10^−2^) from lowest retted (−) to highest retted (+) level. This double positive impact is even more pronounced in the rubbery state, where a 45% increase in E’ was observed between retting (−) and (+) at 130 °C. Yang et al. also noted a similar trend using kenaf fibers mixed with PBAT-PHBV copolymer matrix composite [32]. As was highlighted for the static mechanical results, we also noticed the positive impact of retting on the viscoelastic properties. These findings could be associated to fiber individualization and a cleaner contact surface, allowing both better adhesion and stress transfer. However, the analysis of the loss modulus curves also showed loss modulus peaks between 60 and 110 °C. These peaks are located, respectively, on setup 1 (+) and 3 (+) at 81.3 and 93.2 °C for values of 939.7 and 974.6 MPa, respectively. The higher temperature of the E” peak could be explained by the different processing step between setups 1 and 3. It has been shown by Wu et al. that a more cross-linked structure led to an increase in the alpha relaxation temperature by more constricted macromolecular chains [33].

**Figure 7:**
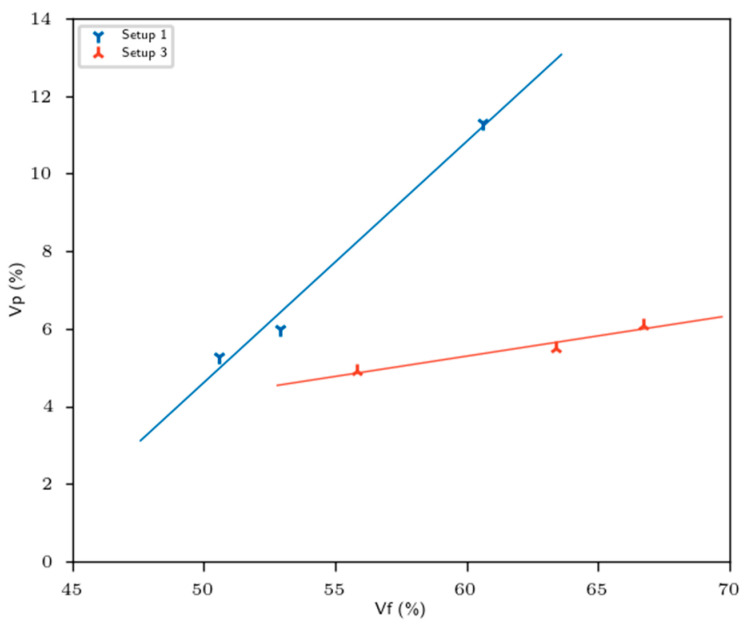
Variation of porosity rate in function of fiber rate.

Regarding the mechanical behavior, we noticed that Young moduli (E1 and E2) gradually increased with retting. The latter had a positive impact on the mechanical effective performance (normalized, see Figure 5). The longer the retting process, the higher the mechanical efficiency of reinforcement, mainly attributed to fiber individualization (see Figure 9). The best compromise in terms of mechanical performance was reached for both long retting and setup 3. Moreover, the porosity rate, which is one of the keys of composite performances, decreased with the retting and reached lower value at setup 3 (+), as illustrated in Figure 7. Consequently, the higher specific surface of the fiber–matrix interface was obtained for setup 3 (+). Nevertheless, the higher values of fiber modulus prove that the efficiency of reinforcement is not optimal for our composites.

### 3.4. Biochemical Analysis

**Figure 8:**
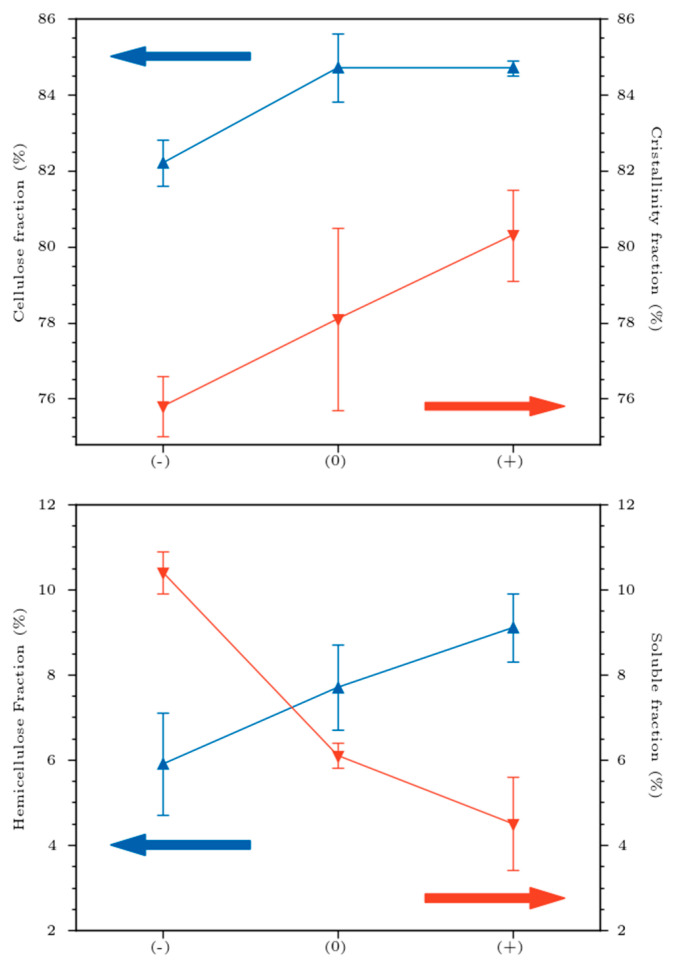
Biochemical fractions (cellulose, hemicellulose, solubles) and crystallinity variations in technical flax fibers. Red and blue arrows indicate the axis for red and blue line.

**Figure 9:**
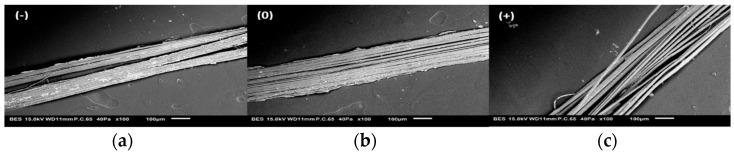
SEM pictures of flax technical fibers (**a**) retting (−), (**b**) retting (0), (**c**) retting (+), (magnification ×100).

Considering the biochemical results (Figure 8), the variation in mechanical properties could be associated to the biochemical alteration of the technical fibers (loss of soluble compounds, i.e., degradation of middle lamella), which led to the individualization of the elementary fibers (Figure 9), consequently reducing the rigidity of technical fibers. In fact, and from a morphological perspective, the cohesion of inter-bundle fibers occurred due to the presence of middle lamella, rich in soluble parts. Increasing the retting level favors the fiber individualization [34]. This phenomenon allowed a cleaner and smoother surface, but slightly affected their final mechanical properties. As is known, the soluble fraction of the fibers was degraded during the retting stage, which indirectly affected the proportion of the different fractions. Furthermore, we noticed that the crystallinity index varied significantly from 75.8 for flax (−) to 80.3% for flax (+), with *p*-value = 1.98 × 10^−2^. Zafeiropoulos et al. [35] obtained a crystallinity index of 70.1 and 71.6%, respectively, for flax fibers washed with 5% caustic soda and dew-retted. Since cellulose is the major contributor to the crystalline fraction of the fiber and the cellulose fraction increased with retting, a direct correlation can be established between the cellulose fraction increase and the crystalline index. These observations are in agreement with those of other studies [36,37]. The variations in chemical composition were also confirmed by spectrophotometric monitoring of the color transition (saturation and brightness) of the technical flax fibers [38].

## 4. Conclusions

In this work, we investigated the retting effect on the mechanical performances of flax/epoxy biocomposite materials manufactured by thermocompression. From a biochemical approach, the retting tends to decrease the soluble elements of the fibers and increase cellulose content. However, hemicellulose and lignin do not seem to be impacted by the retting step. The crystallinity index seems also to increase with retting, which is an expected result, as retting positively affects the cellulose content. The biochemical alteration of flax specimens induced poor mechanical results. The ultimate specific elastic modulus undergoes a drop of −37% for the theoretical elementary fiber and 40% for the theoretical technical fiber when the retting increases (−) to (+). The specific tensile strength decreased by −13% and −53% for the theoretical elementary fiber and the technical fiber, respectively. The degradation of the middle lamella, mostly made of pectin, explains the loss of elasticity properties due to retting. The middle lamella degradation decreased the internal cohesion between the fibrils and consequently affected the elasticity and rigidity performances of technical flax fibers. From a mechanical perspective, advanced retting coupled with setup 3 leads to optimal and highly specific mechanical performance, synonymous with good correlation between high cellulose fraction, high crystallinity and mechanical properties. Performing a normalization of E1 modulus according to the fiber volume, the retting effect was particularly pronounced. Switching from retting (−) to (+) improved the elasticity moduli by 40%. Moreover, the higher the fiber content, the higher the porosity rates (Vp). The same trend was also deduced for viscoelastic behavior. With optimal conditions (retting (+) and improved processing setup 3), the storage modulus was also significantly improved, confirming the efficiency of retting (+)/setup 3 conditions at different scales of mechanical performances. Processing parameters strongly affect the conversion rate of the epoxy monomers and up to 90% of monomer conversion leads to a restriction on the macromolecular chains. Further viscoelastic and wettability analysis will be necessary to better understand the matrix/reinforcement interaction and quality.

## Figures and Tables

**Table 1:** Process parameters of the obtained biobased composite.

	Retting	Times (Min)	Tmax (°C)	P_piston_ (Bars)	P_mold_ (Bars)
1	(−)	370	140	50	2.8
1	(0)	370	140	50	2.8
1	(+)	370	140	50	2.8
3	(−)	130	160	50	2.8
3	(0)	130	160	50	2.8
3	(+)	130	160	50	2.8

(−) under-retted, (0) nominally retted for textile applications, (+) over-retted. (Time) is the total processing duration, (Tmax) is the temperature during processing, (P_piston_), is the direct pressure applied by the hydraulic system, (P_mold_), is the effective pressure applied to the material.
